# Changes in Sociodemographic and Disease Prevalence Among Five Birth Cohorts of Older Latinos

**DOI:** 10.1093/geroni/igab046.1924

**Published:** 2021-12-17

**Authors:** Catherine Garcia, Jennifer Ailshire

**Affiliations:** 1 University of Nebraska - Lincoln, Lincoln, Nebraska, United States; 2 University of Southern California, Los Angeles, California, United States

## Abstract

Latinos are often treated as an amalgamated group without respect to Latinos' composition included in sampling designs in different periods. This matters because the Latino population is continuously changing over time with respect to migration patterns, socioeconomic status, sociocultural characteristics, and geographic dispersion across the U.S., which may influence disease patterns in later life. We use data from the Health and Retirement Study and the National Health Interview Survey to investigate changes in older Latinos' composition by examining five birth cohorts. Results indicate that there have been significant demographic and health changes over time among older Latinos, with later-born cohorts more racially and ethnically diverse, more educated, and exhibiting a higher prevalence of hypertension, diabetes, and obesity. Understanding these shifting dynamics is imperative for crafting strategies and public policies that meet this group's health needs, reduce the cost of health care, and increase the quality of life for older Latinos.

